# Case Report: Popliteal arterial tumor embolus as the initial presentation of EGFR-mutant lung adenocarcinoma with pulmonary-vein invasion

**DOI:** 10.3389/fonc.2025.1731314

**Published:** 2025-12-05

**Authors:** Yongchun Hong, Xinjing Ding, Jianghua Ding

**Affiliations:** 1 Cardiology Department, Jiujiang University Affiliated Hospital, Jiujiang, China; 2 Hematology & Oncology Department, Jiujiang University Affiliated Hospital, Jiujiang, China

**Keywords:** popliteal artery, tumor embolus, EGFR-mutant lung adenocarcinoma, pulmonary-vein invasion, initial presentation

## Abstract

Thrombotic events represent a common complication and the second leading cause of mortality among patients with non-small cell lung cancer (NSCLC). Nonetheless, it is exceedingly rare for thrombosis to manifest initially in atypical locations, such as the popliteal artery, in individuals with NSCLC. In this report, we present a case involving an elderly patient with NSCLC who initially exhibited acute thrombosis of the popliteal artery in the left limb. The patient underwent a salvage arterial thrombectomy followed by a transfemoral amputation. Pathological examination revealed that the thrombus was a tumor embolus. A chest CT scan identified a mass in the right lower lung with a maximum diameter of 2.8 cm, along with multiple pulmonary nodules and a clot in the right inferior pulmonary vein. Pathological analysis of the lung mass confirmed the presence of lung adenocarcinoma, thereby verifying that the tumor embolus originated from NSCLC. The patient subsequently received treatment with osimertinib, resulting in partial remission. In instances of thrombosis occurring in uncommon sites, it is crucial to consider the potential for tumor thrombus.

## 1 Introduction

The global incidence and mortality rates of lung cancer are the highest among all cancers, with non-small cell lung cancer (NSCLC) representing over 85% of cases (1). In patients with advanced NSCLC, clinical manifestations are predominantly characterized by either local invasion-such as cough, hemoptysis, and chest pain-or by metastasis and compression, leading to symptoms like bone pain and nerve compression. Notably, thrombophilia is a significant concern in NSCLC, affecting both arterial and venous systems.

Research indicates that approximately 10% of cancer-related deaths are attributable to thrombophilia (2). Thromboembolic events (TEEs) are particularly prevalent in NSCLC patients, with an incidence rate of 13.6%, which include deep vein thrombosis (DVT), pulmonary embolism (PE) and arterial embolism (AE) (3). The mechanisms underlying thrombosis in non-small cell lung cancer (NSCLC) are complex. They primarily involve cancer-associated hypercoagulability, endothelial damage induced by treatment, and the pro-thrombotic effects of specific genetic mutations (4, 5). Of those, oncogenic driver genes are reported to be closely associated with thrombosis. The presence of ALK/ROS rearrangements is associated with the thrombosis risk of threefold to fourfold increase compared to EGFR-mutant NSCLC (6). Recently, the angiogenesis inhibitors (e.g. bevacizumab) have increased the incidence of arterial thrombosis (2%~4.4%) in NSCLC patients (7, 8).

However, it is rare for the patients with NSCLC to present with artery thrombus as the first manifestation, especially in the uncommon site of popliteal artery. We herein report a patient with NSCLC initially diagnosed as an acute popliteal artery thrombus in the left leg, which was subsequently confirmed to be a tumor thrombus. Unfortunately, the patient missed the ideal time for treatment of thrombolysis and thrombectomy, resulting in having to undergo limb amputation. The uncommon clinical diagnosis warrants greater attention from oncologists.

## 2 Case presentation

### 2.1 Patient information

On Jan. 18, 2024, a 73-year-old male patient presented to the vascular surgery clinic with a two-day history of sudden onset numbness and pain in his left lower extremity. The patient had a 41-year history of smoking and a six-year history of hypertension. He had no history of rheumatic heart disease. Over the past six years, the patient has received standardized antihypertensive treatment. The patient reported experiencing a mild cough, but denied hemoptysis or chest pain. On physical examination, the patient was conscious, alert, and exhibited normal vital signs. Blood pressure measurements indicated a modest elevation at 150/95 mmHg. Peripheral arterial pulses were absent in the left popliteal, posterior tibial, and dorsalis pedis regions; however, pulses in the right limb were normal.

### 2.2 Diagnosis and therapy

**Figure 1:**
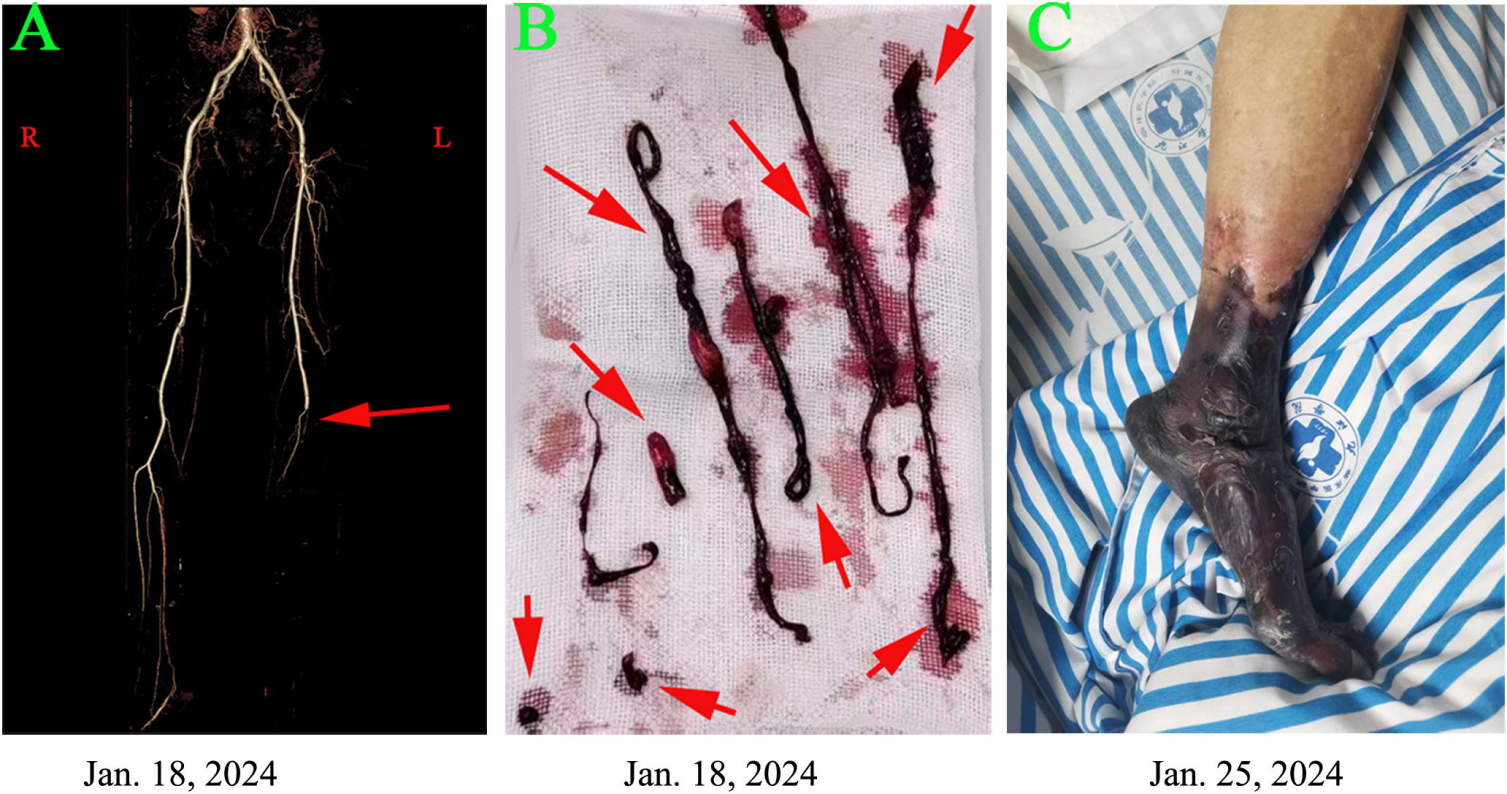
Clinical and imaging findings are presented in the patient with a popliteal artery tumor thrombus as follows: **(A)** Computed tomography angiography on January 18, 2024, revealed complete occlusion distal to the left popliteal artery. **(B)** A salvage percutaneous thrombectomy was performed on the same day, successfully retrieving multiple thrombi. **(C)** Despite these interventions, severe limb ischemia progressed, leading to gangrene in the left leg by January 25, 2024.

Emergency computed tomography (CT) angiography revealed complete obliteration distal to the left popliteal artery (**Figure 1A**). The arterial duplex examination of the contralateral limb demonstrated normal findings. Echocardiographic assessment of the heart revealed no evidence of intracardiac thrombus, vegetative lesions, or nonbacterial thrombotic endocarditis (NBTE). Laboratory analysis indicated elevated levels of D-dimer and fibrinogen, measured at 498 ng/mL and 7.3 g/L, respectively. The thrombin time (TT) and prothrombin time (PT) were recorded at 15.2 seconds and 10.9 seconds, respectively. Platelet count was within normal limits at 230 × 10^9/L. These findings suggest a hypercoagulable state. Consequently, the patient was initiated on anticoagulation therapy with nadroparin calcium at a dosage of 4100 IU per day.

According to the guidelines for acute arterial embolization of the lower extremities, the optimal timeframe for intervention, specifically arterial thrombectomy, is within 8 to 12 hours following the initial clinical presentation (9). In the present case, the interval from symptom onset to diagnosis was approximately 48 hours. Although the optimal window for interventional treatment was missed, the patient underwent salvage arterial thrombectomy on January 18, 2024 (**Figure 1B**). Despite this intervention, the occlusion continued to progress. By January 25, 2024, the patient’s condition had advanced to Rutherford class VI. Consequently, a transfemoral amputation was performed on January 26, 2024 (**Figure 1C**). Postoperatively, the patient was administered anticoagulation therapy with nadroparin calcium and received best supportive care. Fortunately, the patient experienced a smooth recovery following the surgery.

**Figure 2:**
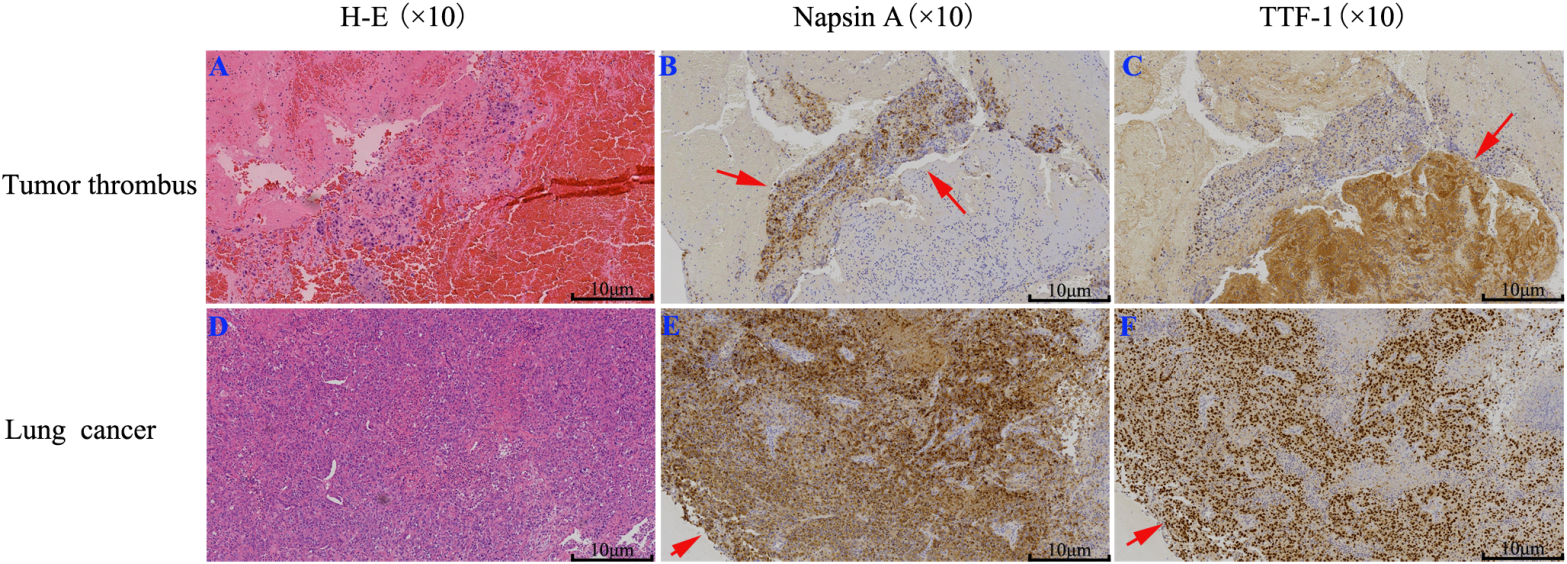
IHC staining of lung cancer and tumor thrombus: **(A, D)** H-E staining; **(B, E)** Napsin A; **(C, F)** TTF-1. The results suggest the tumor thrombus originated from lung adenocarcinoma.

**Figure 3:**
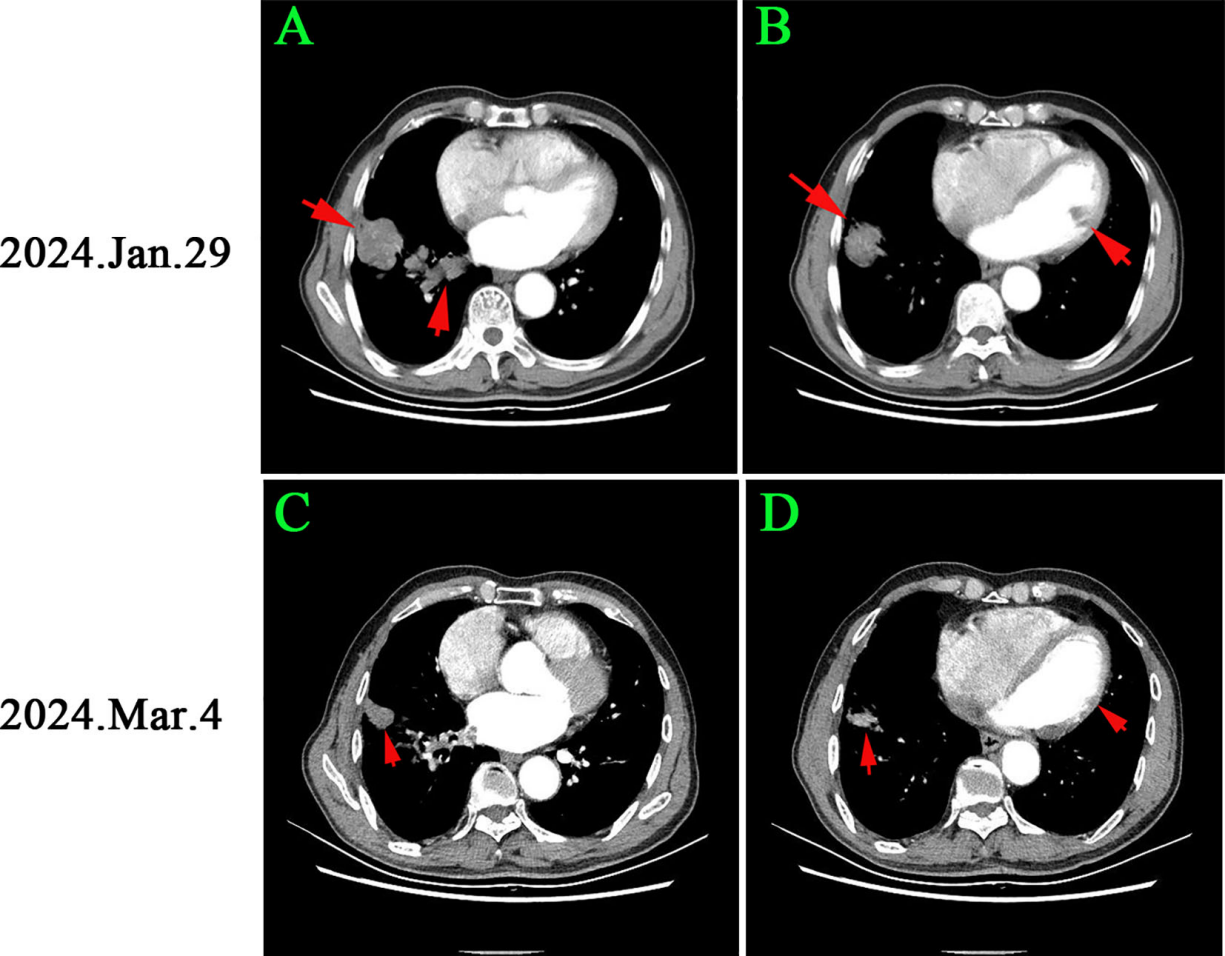
Chest CT changes before and after osimertinib treatment: **(A, B)** Initial imaging showed a 2.8 cm right lower lung mass and multiple pulmonary nodules, with a clot in the right inferior pulmonary vein. **(C, D)** After 1 month of osimertinib, the lung cancer lesion reduced to 1.2 cm, and the pulmonary nodules significantly decreased, with no filling defects in the vein. According to RECIST criteria, the patient achieved part remission (PR).

On January 28, 2024, the thrombus was identified as malignant (**Figures 2A–C**). Notably, the levels of carcinoembryonic antigen (CEA) and cytokeratin-19 fragment (CYFRA 21-1) were markedly elevated, measuring 65.2 ng/mL and 30.8 ng/mL, respectively. Subsequently, computed tomography (CT) scans of the chest and abdomen were conducted on January 29, 2024. The abdominal scan revealed no masses, while the chest CT identified a mass in the right lower lung with a maximum diameter of 2.8 cm, accompanied by multiple pulmonary nodules (**Figure 3A**). Additionally, filling defects were observed in the right inferior pulmonary vein, suggestive of a thrombus (**Figure 3B**). On January 30, 2024, the patient underwent a percutaneous transthoracic needle biopsy. Pathological examination on February 1, 2024, confirmed the presence of lung adenocarcinoma, as indicated by positive staining for Napsin A and thyroid transcription factor 1 (TTF-1) (**Figures 2D–F**). These findings collectively suggest that the tumor embolus originated from lung adenocarcinoma.

### 2.3 Follow-up and clinical outcome

On Feb. 3, 2024, genetic testing identified an L858R mutation in the EGFR at exon 21. Following the NCCN guidelines (2024.V1), osimertinib was prescribed as the preferred treatment for advanced NSCLC with this mutation, and the patient began an 80 mg daily dosage. Anticoagulation therapy with nadroparin calcium was also continued daily.

**Figure 4:**
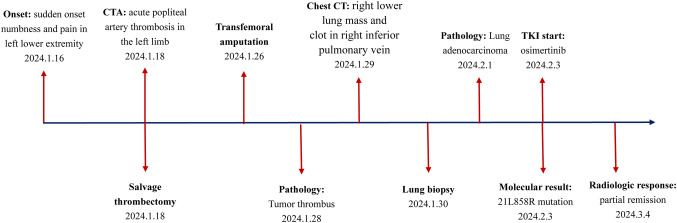
The detailed timeline of the patient’s clinical course.

On March 4, 2024, CEA and CYFRA211 levels decreased to 15.2 ng/ml and 9.8 ng/ml. D-dimer and Fib levels were 1.3 ng/ml and 2.6 g/L. TT and PT were 13.5 and 12.9 seconds (**Figure 3C**). A chest CT scan showed a 57.1% reduction in the right lower lung tumor and significant reduction in nearby nodules. Filling defects in the right inferior pulmonary vein disappeared (**Figure 3D**). The patient achieved partial remission (PR) per RECIST criteria. Antithrombotic treatment was stopped due to improved hypercoagulable state, and osimertinib treatment continued. The patient has been monitored with no tumor recurrence (**Figure 4**).

## 3 Discussion

Patients with cancer generally exhibit a significantly elevated risk of venous thromboembolism (VTE) compared to case-control subjects without cancer. VTE is a significant contributor to morbidity and mortality among cancer patients, primarily encompassing deep venous thromboembolism (DVT) (4% to 20%) and pulmonary embolism (PE) (2% to 5%) (10). VTE significantly impacts the quality of life of cancer patients and can occasionally manifest as life-threatening complications. Moreover, elderly patients are at an increased risk of VTE and tend to experience poorer outcomes compared to younger adults. Consequently, accurate assessment of VTE risk is essential to prevent mortality in high-risk populations. In addition to the common types of DVT and PE, the incidence of arterial thrombosis is increased in patients with malignancies.

In the context of non-small cell lung cancer (NSCLC), there are some instances of arterial thrombosis have been documented. These include acute coronary artery thrombosis following diagnosis (11), chemotherapy-induced acute aortic thrombosis (8, 9), and acute lower extremity arterial thrombosis linked to osimertinib-induced erythrocytosis (12). Also, risk of thromboembolism is closely associated with the molecular subtypes in patients with NSCLC. A cohort study revealed that the highest incidences of thromboembolism were observed in patients with ALK-mutant NSCLC (43.5%) followed by patients with EGFR-mutant cancers (21.2%) and wild-type cancers (17.2%). Patients who experienced thromboembolism had worse overall survival (13). Wang HY et al. reported that 43.2% of thromboembolic events were arterial in the EGFR-mutant NSCLC. On the contrary, venous thromboembolic events mainly occurred in the ROS1 and ALK cohorts (14). Furthermore, Watanabe H et al. investigated that multiple organ infarctions were attributed to an aortic thrombus in a lung cancer patient with a BRAF mutation (15).

Notably, some patients exhibit tumor embolism prior to the clinical diagnosis of the primary malignancy. For instance, Sato A et al. described a case of acute arterial occlusive disease caused by a tumor thrombus originating from lung metastasis of breast cancer (16). Similarly, Zhao Y et al. identified a rare clinical occurrence of acute upper limb ischemia resulting from metastatic tumor thromboembolism (17). In the present manuscript, the patient initially presented with acute numbness and pain in the left leg. The clots in the left popliteal artery were tumor emboli originating from lung cancer. The underlying physiological pathway involves the flow of blood from the pulmonary veins into the left atrium and subsequently into the left ventricle. The left ventricle then propels blood into the aorta, which distributes it throughout the systemic arteries, including the left popliteal artery. The early diagnosis of lung cancer was delayed due to the absence of typical symptoms including hemoptysis or chest pain, as well as the thrombosis occurring in the uncommon location of the popliteal artery. Fortunately, the patient accepted osimertinib treatment for carrying EGFR 21L858R mutation, and now still in part remission.

In the case of the present patient, several factors contribute to the development of tumor thrombus. Firstly, the patient was diagnosed at an advanced stage of the disease, which is recognized as an independent prognostic factor linked to an increased thromboembolic risk in non-small cell lung cancer (NSCLC) (14). Secondly, the patient demonstrated a state of hypercoagulability, as indicated by elevated levels of D-dimer. Additionally, the presence of pulmonary venous thrombosis significantly heightens the risk of tumor embolism. It has been reported that overall thrombotic events occur in 59.4% of NSCLC cases, with 53.6% of these events being associated with tumor emboli or tumor vascular infiltration (18). Finally, the patient exhibited the 21L858R mutation, which is associated with an elevated risk of arterial thrombus formation. A retrospective study indicated that NSCLC patients with EGFR mutations experience a higher incidence of arterial thrombotic events, whereas those with ALK-positive lung cancer are more prone to venous events (14). Furthermore, it has been suggested that EGFR mutations may upregulate coagulation proteins, such as fibrinogen, thereby increasing the risk of thrombus formation (13).

In conclusion, patients with lung cancer are recognized to have an elevated risk of developing thrombosis. Notably, the patient did not regard the mild cough as serious, resulting in delayed diagnosis of lung cancer. In cases of thrombus located in rare anatomical sites, particularly for elderly patients, it is essential to consider the possibility of tumor thrombus.

## Data Availability

The original contributions presented in the study are included in the article. Further inquiries can be directed to the corresponding author.
